# A comprehensive integration of data on the association of ITPKC polymorphisms with susceptibility to Kawasaki disease: a meta-analysis

**DOI:** 10.1186/s12920-025-02121-8

**Published:** 2025-03-20

**Authors:** Atefeh Habibi, Hanieh Talebi, Reza Bahrami, Mohammad Golshan-Tafti, Amirhossein Shahbazi, Seyed Alireza Dastgheib, Azadeh Tahooni, Maryam Vafapour, Heewa Rashnavadi, Melina Pourkazemi, Maryam Yeganegi, Elnaz Sheikhpour, Hossein Neamatzadeh

**Affiliations:** 1 Department of Pediatrics, Hakim Children Hospital, Tehran University of Medical Sciences, Tehran, Iran; 2 Clinical Research Development Unit, Fatemieh Hospital, Hamadan University of Medical Sciences, Hamadan, Iran; 3 Neonatal Research Center, Shiraz University of Medical Sciences, Shiraz, Iran; 4 Department of Pediatrics, School of Medicine, Islamic Azad University of Yazd, Yazd, Iran; 5 Student Research Committee, School of Medicine, Ilam University of Medical Sciences, Ilam, Iran; 6 Department of Medical Genetics, School of Medicine, Shiraz University of Medical Sciences, Shiraz, Iran; 7 Department of Cardiology, Firoozgar Hospital Research Center, Iran University of Medical Sciences, Tehran, Iran; 8 Department of Pediatrics, Firoozabadi Clinical Research Development Unit, Iran University of Medical Sciences, Tehran, Iran; 9 Student Research Committee, School of Medicine, Tehran University of Medical Sciences, Tehran, Iran; 10 Student Research Committee, School of Medicine, Iran University of Medical Sciences, Tehran, Iran; 11 Department of Obstetrics and Gynecology, Iranshahr University of Medical Sciences, Iranshahr, Iran; 12 Hematology and Oncology Research Center, Shahid Sadoughi University of Medical Sciences, Yazd, Iran; 13 Mother and Newborn Health Research Center, Shahid Sadoughi University of Medical Sciences, Yazd, Iran

**Keywords:** Kawasaki disease, ITPKC, Polymorphism, Coronary artery, Inflammation

## Abstract

**Background:**

This study aims to conduct a comprehensive meta-analysis of existing research to define clear associations between variations in the ITPKC gene and the risk of developing Kawasaki disease (KD).

**Methods:**

A comprehensive search was conducted across multiple databases, including but not limited to PubMed, Scopus, EMBASE, and CNKI, up to June 1, 2024, to gather relevant information. This search utilized keywords and MeSH terms related to hyperbilirubinemia and genetic factors. The inclusion criteria encompassed original case-control, longitudinal, or cohort studies. Correlations were analyzed as odds ratios (ORs) with 95% confidence intervals (CIs) using Comprehensive Meta-Analysis software.

**Results:**

Eighteen case-control studies with 5,434 KD cases and 9,419 controls were analyzed. Of these, ten studies assessed 3,129 KD cases and 6,172 controls for the rs28493229 variant, four examined 1,039 cases and 1,688 controls for the rs2290692 variant, two focused on 595 cases and 820 controls for the rs7251246 variant, and two investigated 671 cases and 739 controls for the rs10420685 variant. Results showed a significant association between the rs28493229 polymorphism and increased KD risk across all five genetic models. Subgroup analysis indicated this polymorphism correlates with KD susceptibility in Asians but not in the Chinese population. In contrast, no associations were found between the rs2290692, rs7251246, and rs10420685 polymorphisms and KD risk.

**Conclusions:**

Our pooled data indicate a significant association between the ITPKC rs28493229 polymorphism’s minor allele and an increased risk of developing KD, suggesting this variant may enhance susceptibility. Conversely, SNPs rs2290692, rs7251246, and rs10420685 do not demonstrate a statistically significant relationship with KD.

**Supplementary information:**

The online version contains supplementary material available at 10.1186/s12920-025-02121-8.

## Introduction

Kawasaki disease (KD; OMIM 300530) is characterized by inflammation of medium-sized blood vessels [1, 2]. Over recent decades, KD has become the primary cause of acquired heart disease in children in East Asian, European, and North American regions [3, 4]. It mainly impacts children under 5 years old and is thought to be triggered by an abnormal immune response to an infectious agent in those with a genetic predisposition [5, 6]. Population-based data adjusted for age shows that children under five are mainly affected by KD, with males having a higher prevalence [7, 8]. The prevalence of KD exhibits variations across different geographical regions and time periods. For instance, from 2006 to 2021, England recorded a KD prevalence that surpassed clinician reports, with an incidence rate of 8.9 cases per 100,000 person-years among children under 5 years old [9]. In Nova Scotia, Canada, the annual KD incidence was 29.6 cases per 100,000 children under 5 [10]. Japan experienced a 35.6% decrease in diagnosed KD cases in 2020 compared to the previous year, potentially linked to the impact of the COVID-19 pandemic [11]. Switzerland documented an incidence rate of 3.1 cases per 100,000 per year among children under 17, and 8.4 cases per 100,000 per year among children under 5 [12]. A nationwide registry study conducted in Iran from 2007 to 2019 identified 1,682 cases of KD. The yearly incidence of the disease ranged from 2.62 to 3.03 cases per 100,000 population between 2015 and 2019, with a male-to-female ratio of 1.46 [13]. KD diagnosis is confirmed by fever lasting at least 5 days and at least four signs of inflammation in the skin and mucous membranes. Severe KD requires prompt treatment escalation, with coronary artery lesions (CAL) being key prognostic indicators [14]. However, incomplete Kawasaki disease (IKD) presents challenges in diagnosis and management due to the lack of specific markers and clear diagnostic criteria [15]. Standard KD treatment involves intravenous immunoglobulin (IVIG) and acetylsalicylic acid (ASA/aspirin). IVIG is the preferred therapy and has been effective in reducing coronary artery abnormalities (CAAs) compared to ASA. Higher IVIG doses are associated with a lower risk of CAA formation compared to medium- or low-dose regimens. Furthermore, IVIG treatment not only reduces fever duration but may also decrease the need for additional therapeutic interventions [14, 16].

Recent investigations have shed light on the intricate processes underlying immune activation in KD. Nonetheless, the precise origins of KD remain elusive. It is postulated that KD stems from an atypical inflammatory reaction triggered by a single or multiple infectious organisms or toxic substances in individuals with a genetic predisposition [17]. Genome-wide association studies (GWAS) have been utilized in order to detect genetic variants that are linked to the susceptibility of KD as well as the response to treatment [18, 19]. Various investigations have identified significant rare variant genes, such as ITPKC, CASP3, BLK, MYH14, FCGR2A and RBP3, which may potentially be associated with the susceptibility of KD [20, 21]. Additionally, whole genome sequencing (WGS) has been employed to identify predictors of non-response to the standard therapy for KD, IVIG treatment. WGS studies have validated polymorphisms in genes previously implicated in the IVIG pathway and have also discovered multiple novel SNPs in different gene regions that could have a role in IVIG response [22]. However, GWAS studies have encountered difficulties, including the “missing heritability” issue, and further functional studies are required to establish a precise connection between risk alleles and the KD phenotype [23, 24]. High-throughput DNA sequencing technology, such as WGS, possesses the potential to offer more extensive genomic information, thereby enhancing our understanding of the etiology and treatment of KD. Genetic factors, consisting of mutations in genes involved in T lymphocyte activation, have been found to contribute to IVIG resistance in KD patients [25].

As an inositol-1,4,5-trisphosphate (IP3) kinase, ITPKC is a crucial second messenger that triggers calcium release from the endoplasmic reticulum and sarcoplasmic reticulum [26]. Alongside other kinases, ITPKC phosphorylates IP3 to convert it into inositol-1,3,4,5-tetrakisphosphate (IP4), thereby impeding the IP3-mediated calcium discharge from the cell. The influx of calcium initiates the Ca2+/NFAT pathway, setting off signaling cascades that activate T cells and immune cells, potentially leading to immune disorders or autoimmune reactions [27]. In 2008, Onouchi and colleagues identified the ITPKC gene (ID:80271) as associated with KD in Japanese and American populations, located on chromosome 19q13.2. By performing linkage disequilibrium (LD) mapping in this genomic region, they detected a specific single nucleotide polymorphism (SNP) within the ITPKC gene that was linked to susceptibility to KD and increased risk of CAL [28]. ITPKC functions as a negative regulator of the Ca2+/NFAT signaling pathway, with the C allele potentially exacerbating immune responses in KD [29]. Moreover, different ITPKC variations could amplify T cell activation and interleukin-2 (IL-2) expression, thereby intensifying the pro-inflammatory response of T cells during the acute phase, potentially increasing susceptibility to KD and disease severity [30]. Research indicates that functional variations in the ITPKC gene increase the risk of IVIG resistance and are linked to the concurrent development of CAL in KD patients. The unclear link between ITPKC polymorphisms and KD risk remains due to insufficient large-scale, well-designed studies examining this relationship [31]. A meta-analysis can enhance statistical power by combining data from various studies, allowing for a more precise assessment of the impact of genetic polymorphisms on disease susceptibility [32, 33].

This meta-analysis investigates the link between ITPKC polymorphisms—specifically rs2290692, rs28493229, rs7251246, and rs10420685—and susceptibility to KD using various genetic models. It particularly focuses on rs2290692 due to prior associations with KD susceptibility. Despite ITPKC’s known role in the Ca2+/NFAT signaling pathway linked to immune responses, findings on these associations have varied across different populations [34, 35]. Previous studies on ITPKC polymorphisms, including rs28493229, have produced conflicting results [34, 36, 37], highlighting the need for a thorough review to clarify how these genetic variations influence disease susceptibility. By aggregating data from multiple studies, this analysis improves statistical power, enabling the detection of subtle effects often overlooked in smaller studies. Ultimately, this research aims to provide updated insights into the relationships between these polymorphisms and KD susceptibility, enhancing our understanding of the disease’s pathogenesis.

## Materials and methods

### Literature search strategy

This study conducts an in-depth literature review across a variety of databases, including MEDLINE, PubMed, PubMed Central (PMC), Europe PubMed Central (Europe PMC), Scopus, Cochrane Library, Google Scholar, Web of Science, Elsevier, Cumulative Index to Nursing and Allied Health Literature (CINAHL), ResearchGate, ClinicalTrials.gov, SciELO, MedNexus, MedRxiv, Chinese Biomedical Database (CBD), Chinese National Knowledge Infrastructure (CNKI), Wanfang Data Company, Chaoxing, Circumpolar Health Bibliographic Database (CHBD), China/Asia On Demand (CAOD), Indian Citation Index (ICI), Chinese Medical Citation Index (CMCI), Semantic Scholar, Egyptian Knowledge Bank (EKB), VIP Information Consultancy Company (VIP), Chinese Medical Current Contents (CMCC), and Weipu Periodical Database, up until June 1, 2024. The objective of this review is to systematically evaluate the connection between variations in the ITPKC gene and susceptibility to Kawasaki disease. Additionally, relevant citations from the included studies were examined manually. The investigation employed a combination of MeSH terms and keywords related to Kawasaki disease, ITPKC, and genetic mutations, specifically including terms such as “Kawasaki Disease,” “KD,” “Incomplete Kawasaki disease,” “coronary artery abnormalities,” and “Mucocutaneous Lymph Node Syndrome,” along with “Inositol 1,4,5-trisphosphate 3-kinase C,” “ITPKC,” “IP3KC,” “IP3-3KC,” as well as terms related to genes, single-nucleotide polymorphisms (SNPs), genotypes, alleles, variations, and mutations. Furthermore, references from relevant reviews and reputable publications were assessed to identify additional potential sources. In cases where multiple studies were attributed to the same author(s) with duplicated or overlapping data, the meta-analysis incorporated the study with the largest sample size or the most recently published research.

### Inclusion and exclusion criteria

The studies were meticulously chosen to ensure their relevance and quality, and those meeting the established criteria were deemed eligible for inclusion in the analysis. The inclusion criteria were: (1) research studies using a case-control or cohort design; (2) investigations examining the correlation between ITPKC polymorphisms and susceptibility to KD; (3) studies providing sufficient and accessible data for calculating an odds ratio (OR) and a 95% confidence interval (CI). Conversely, studies were excluded if they: (1) involved animal subjects or were experimental studies conducted in vitro; (2) lacked complete genotype frequency data; (3) were based on linkage or family-based analyses, such as those involving siblings, twins, and parent-trios; (4) were abstracts, case reports, commentaries, editorials, conference papers, reviews, proceedings, or meta-analyses; or (5) were duplicates of other studies.

### Data extraction

Two authors independently assessed all eligible articles and collected all required information based on the specified inclusion criteria. In case of any discrepancies, a discussion was held to reach a consensus. If consensus could not be reached, a third researcher was consulted for resolution, with the final decision based on majority vote. Each eligible study included data on the primary author’s name, publication date, country of origin, participants’ ethnic backgrounds (specifically categorized as Asian, Caucasian, African, Hispanic, and Mixed), genotyping methods, source of healthy subjects, total sample size, allele and genotype frequencies of ITPKC variants in healthy subjects and cases, Minor Allele Frequency (MAFs), and Hardy-Weinberg equilibrium (HWE) in healthy controls. This meta-analysis considered separate case-control groups or cohorts within a single publication as individual studies.

### Quality score assessment

The Newcastle-Ottawa Score (NOS) was used to assess the quality of studies in a meta-analysis and evaluate methodological aspects of observational research. This score evaluated case selection, group comparability, and exposure determination, each with eight specific items. Studies with exceptional selection and exposure received one star, while comparability could earn up to two stars. Quality was rated on a nine-star scale, with zero indicating poor quality and nine denoting high quality. Studies scoring seven or more were considered high quality, while those with at least five points were suitable for meta-analysis. Disagreements were resolved through discussion and consensus [38].

### Statistical analysis

Statistical analyses for this study were conducted using Comprehensive Meta-Analysis (CMA) Software version 2.0, developed by Biostat in Englewood, USA, with a significance threshold set at *p* < 0.05 for two-sided tests. The research aimed to investigate the correlation between ITPKC genetic variations and susceptibility to KD, employing ORs along with their corresponding 95% CIs. The significance of pooled ORs was assessed using the Z-test, where *p* < 0.05 indicated statistical significance. Five genetic models were analyzed: allelic (M vs. W), homozygote (MM vs. WW), heterozygote (MW vs. WW), dominant (MM + MW vs. WW), and recessive (MM vs. MW + WW), with ‘M’ denoting the mutant allele and ‘W’ the wild type allele. The Hardy-Weinberg equilibrium (HWE) in the control group was evaluated using the Fisher exact test, with *p* < 0.05 indicating a deviation from HWE [39–41]. A chi-square test was utilized to assess study heterogeneity, with *p* < 0.10 signifying significant heterogeneity, while the I² statistic quantified heterogeneity on a scale from 0 to 100%. In instances of significant heterogeneity, a random-effects model (DerSimonian and Laird method) was applied, whereas a fixed-effects model (Mantel-Haenszel method) was used otherwise [42, 43]. Subgroup analyses based on ethnicity, country, control source, and genotyping methods were performed to explore potential sources of heterogeneity. A one-way sensitivity analysis tested the stability of results by excluding one study at a time, and studies that violated HWE were excluded in a separate sensitivity analysis. Publication bias was assessed using Begg’s funnel plots, where asymmetry suggested potential bias, and Egger’s linear regression test evaluated plot symmetry. If publication bias was identified, the trim-and-fill method was employed to adjust the results.

## Results

### Characteristics of selected studies

**Fig. 1:**
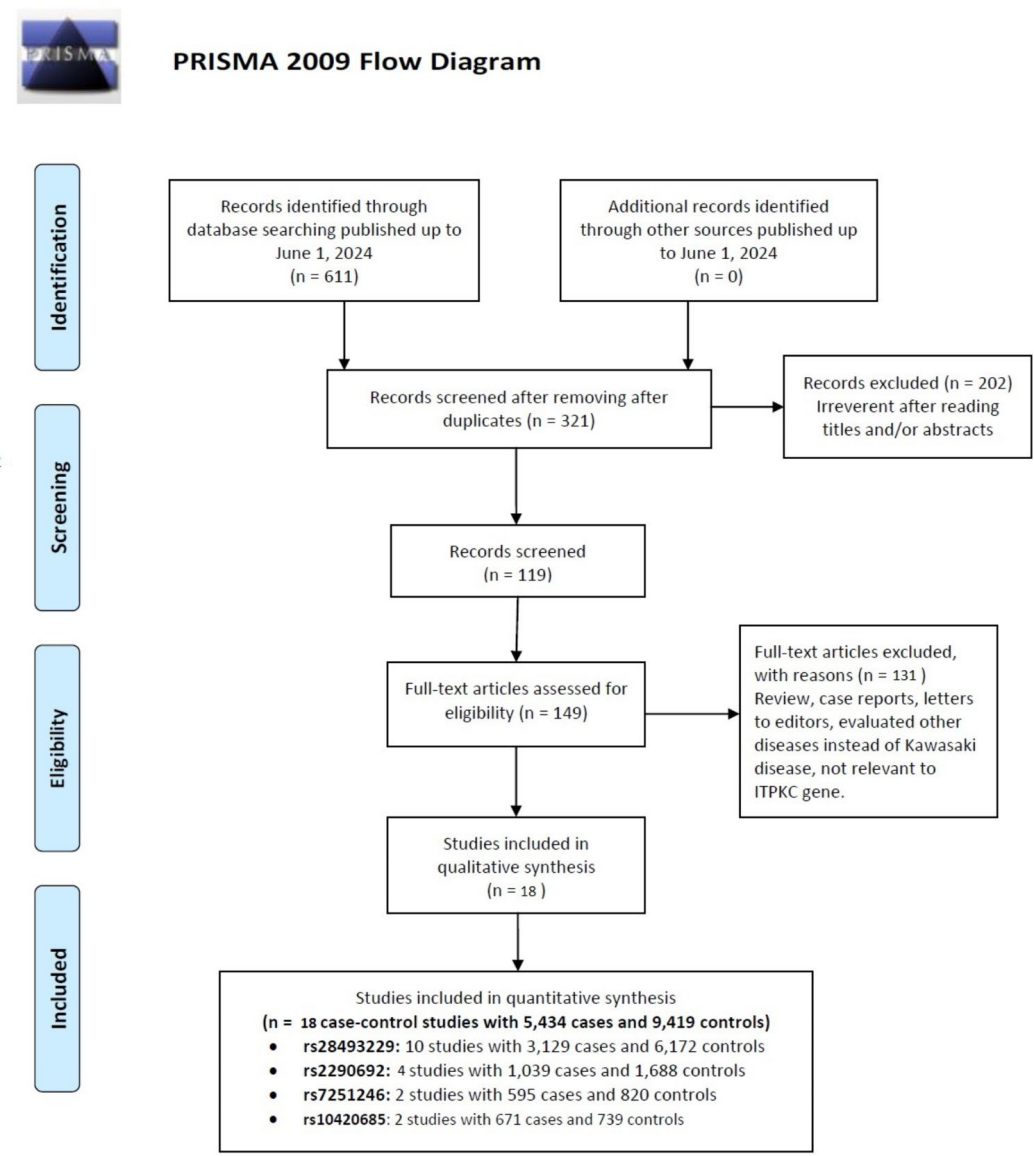
Flowchart illustrating the literature search and selection process

The process of conducting a literature review and selecting relevant articles is depicted in Fig. 1. Initially, the search strategy identified 611 potentially relevant articles. After removing 290 duplicate studies, 321 potentially relevant articles remained. These articles were then screened based on their titles and abstracts, leading to the exclusion of 202 articles that did not meet the predefined inclusion criteria. The remaining 149 articles underwent a full-text review, and an additional 131 studies were excluded for various reasons, such as not reporting the outcome of interest or having an inappropriate study design. Ultimately, 18 case-control studies from 11 independent investigations [28, 34, 35, 44–51], encompassing 5,434 instances of KD and 9,419 controls, were deemed eligible and included in the meta-analysis. Within these, ten studies covered 3,129 cases and 6,172 controls regarding the rs28493229 genetic variant, four studies included 1,039 cases and 1,688 controls on the rs2290692 variant, two studies involved 595 cases and 820 controls on the rs7251246 variant, and two studies involved 671 cases and 739 controls on the rs10420685 variant. Table 1 summarizes the characteristics of studies conducted from 2008 to 2023, highlighting KD case incidence ranging from 17 to 637. The studies encompassed various ethnic groups, with 17 focusing on Asian populations and one on Caucasians, conducted in countries including Japan, China, South Korea, India, and Australia. Four genotyping techniques—TaqMan, direct sequencing, RFLP-PCR, and SNaPshot—were employed to analyze ITPKC polymorphisms. Table 1 also details the allele, genotype, HWE and MAF distributions of these polymorphisms in KD cases and healthy controls.

**Table 1:** Characteristics of studies included in this meta-analysis

Author/Year	Country (Ethnicity)	SOC	Genotyping Methods	Case/Control	KD Cases					Controls					MAFs	HWE	NOS
					**Genotypes**			**Allele**		**Genotypes**			**Allele**				
rs28493229					**GG**	**GC**	**CC**	**G**	**C**	**GG**	**GC**	**CC**	**G**	**C**			
Onouchi 2008	Japan(Asian)	PB	TaqMan	637/1034	376	234	27	986	288	756	249	29	1761	307	0.148	0.126	8
Chi 2010	China(Asian)	NS	TaqMan	385/1158	323	61	1	707	63	1008	147	3	2163	153	0.066	0.327	8
Lin 2011	China(Asian)	NS	Sequencing	280/492	236	43	1	515	45	454	37	1	945	39	0.040	0.787	8
Kuo 2011	China(Asian)	PB	TaqMan	334/1131	282	50	2	614	54	981	142	8	2104	158	0.070	0.256	8
Peng 2012	China(Asian)	HB	PCR-RFLP	223/318	195	27	1	417	29	274	40	4	588	48	0.075	0.078	7
Onouchi 2013	Japan(Asian)	PB	Sequencing	546/938	330	191	25	851	241	662	261	15	1585	291	0.155	0.059	8
Yan 2013	China(Asian)	HB	TaqMan	358/815	315	40	3	670	46	703	107	5	1513	117	0.072	0.673	7
Natividad 2013	Australia(Caucasian)	HB	Sequencing	17/26	16	1	0	33	1	22	4	0	48	4	0.077	0.670	6
Kim 2018	Korea(Asian)	HB	TaqMan	299/210	231	64	4	526	72	178	32	0	388	32	0.076	0.232	7
Bhattarai 2021	India(Asian)	HB	PCR-RFLP	50/50	39	11	0	89	11	42	8	0	92	8	0.080	0.538	6
rs2290692					**GG**	**GC**	**CC**	**G**	**C**	**GG**	**GC**	**CC**	**G**	**C**			
Kuo 2011	China(Asian)	PB	TaqMan	373/551	73	194	106	340	406	122	275	154	519	583	0.529	0.970	8
Peng 2012	China(Asian)	HB	PCR-RFLP	223/318	54	105	64	213	233	116	150	52	382	254	0.399	0.764	7
Kim 2018	Korea(Asian)	HB	TaqMan	299/210	89	143	67	321	277	65	97	48	227	193	0.460	0.309	7
Bhattarai 2021	India(Asian)	HB	PCR-RFLP	50/50	19	25	6	63	37	20	16	14	56	44	0.440	0.013	6
rs7251246					**TT**	**TC**	**CC**	**T**	**C**	**TT**	**TC**	**CC**	**T**	**C**			
Kuo 2011	China(Asian)	PB	TaqMan	374/558	74	197	103	345	403	125	277	156	527	589	0.528	0.922	8
Liu 2023	China(Asian)	HB	SNaPshot	221/262	64	114	43	242	200	80	137	45	297	227	0.433	0.294	7
rs10420685					**AA**	**AG**	**GG**	**A**	**G**	**AA**	**AG**	**GG**	**A**	**G**			
Kuo 2011	China(Asian)	PB	TaqMan	372/529	233	123	16	589	155	332	172	25	836	222	0.210	0.654	8
Kim 2018	Korea(Asian)	HB	TaqMan	299/210	156	116	27	428	170	114	76	20	304	116	0.276	0.169	7

Abbreviations: SOC - source of control; PB - population-based; HB - hospital-based; NS - not stated; PCR - polymerase chain reaction; PCR-RFLP - polymerase chain reaction-restriction fragment length polymorphism; MAF - minor allele frequency; HWE - Hardy-Weinberg equilibrium; NOS - Newcastle-Ottawa Scale

### Quality of included studies

To evaluate the quality of studies in the meta-analysis, we considered several key factors: NOS scores, adherence to HWE, sample size, genotyping methods, and ethnic diversity. NOS scores of 6 to 8 reflect varying quality, with higher scores indicating better participant selection, comparability, and outcome assessment. However, a few studies, particularly those from Natividad (2013) and Bhattarai (2021), received lower scores of 6, suggesting potential limitations in either their sampling methods or control group selection. Adherence to HWE is essential, as studies meeting this criterion suggest a reliable genotype distribution, while violations raise concerns about validity, especially when *p*-values are under 0.05. Larger sample sizes bolster result robustness, as seen in Onouchi 2008 and Kuo 2011, in contrast to the 17 cases in Natividad 2013, which undermine reliability. The choice of genotyping methods affects data quality, with TaqMan assays being more efficient and reliable. Furthermore, ethnic diversity can influence allele frequency and disease associations, highlighting the importance of generalizability. Overall, studies with higher NOS scores and better HWE compliance are considered higher quality, while those with significant HWE deviations or small sample sizes need careful interpretation.

### Quantitative data synthesis

#### rs28493229

**Fig. 2:**
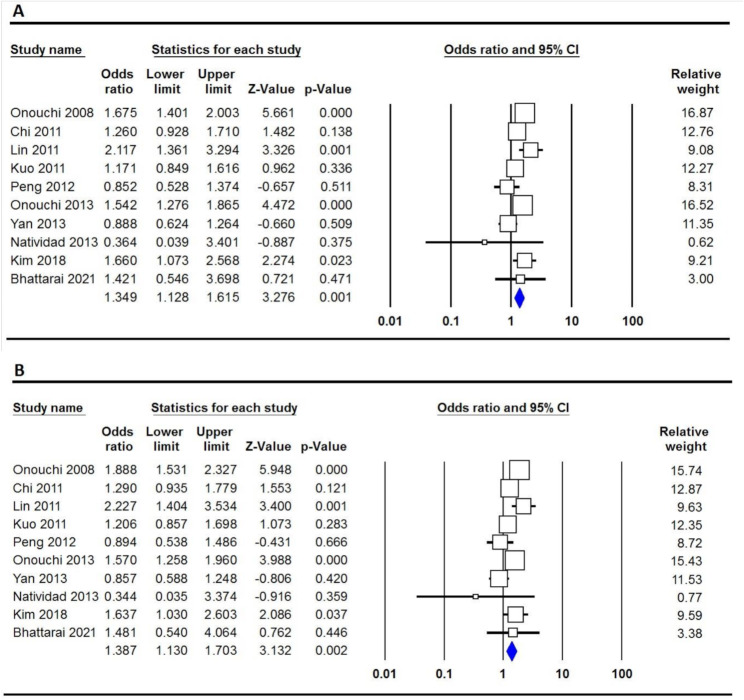
Forest plot showing the association between the ITPKC rs28493229 polymorphism and KD risk in the overall population under the allele model (**A**) and the dominant model (**B**)

Table 2 shows a significant association between the rs28493229 polymorphism and increased susceptibility to KD. The analysis reveals a robust link between the ITPKC polymorphism and KD risk across all five genetic models: allele (C vs. G: OR = 1.349, 95% CI 1.128–1.615, *p* = 0.001, Fig. 2A), homozygote (CC vs. GG: OR = 2.025, 95% CI 1.399–2.929, *p* ≤ 0.001), heterozygote (CG vs. GG: OR = 1.375, 95% CI 1.125–1.681, *p* = 0.002), dominant (CC + CG vs. GG: OR = 1.387, 95% CI 1.130–1.703, *p* = 0.002, Fig. 2B), and recessive (CC vs. CG + GG: OR = 1.767, 95% CI 1.224–2.550, *p* = 0.002). Subgroup analyses among Asian populations showed consistent findings, with a C vs. G OR of 1.361 (95% CI: 1.139–1.627, *p* < 0.001) and a CC vs. GG OR also at 2.025 (95% CI: 1.399–2.929, *p* < 0.001). Additionally, both the CG and CC + CG genotypes versus GG demonstrated ORs of 1.390 (95% CI: 1.139–1.697, *p* < 0.001) and 1.403 (95% CI: 1.145–1.719, *p* < 0.001), respectively. On the other hand, the Chinese subgroup showed no significant associations, with ORs near 1 across all models, suggesting no heightened risk. The source of controls was crucial; population-based studies indicated no significant relationship (C vs. G OR of 1.103, 95% CI: 0.591–2.060, *p* = 0.758), whereas hospital-based studies reported a significant association (C vs. G OR of 1.541, 95% CI: 1.366–1.739, *p* < 0.001; CC vs. GG OR of 2.214, 95% CI: 1.482–3.308, *p* < 0.001). Furthermore, genotyping methods like the TaqMan assay and sequencing showed significant associations across comparisons, notably an OR of 1.493 (95% CI: 1.308–1.705, *p* < 0.001) for C vs. G and 3.248 (95% CI: 1.719–6.136, *p* < 0.001) for CC vs. GG, respectively. Overall, these findings suggest that the ITPKC rs28493229 polymorphism contributes to increased KD risk, especially in Asian and hospital-based populations, while its relevance diminishes in the Chinese subgroup and in population-based studies.

**Table 2:** Summary risk estimates for association of ITPKC rs28493229 polymorphism with KD risk

Subgroup	Genetic Model	Type of Model	Heterogeneity		Odds Ratio				Publication Bias	
			**I** ^**2**^ **(%)**	**P**_**H**_	**OR**	**95% CI**	**Z**_**test**_	**P**_**OR**_	**P**_**Beggs**_	**P**_**Eggers**_
Overall	C vs. G	Random	59.46	0.008	1.349	1.128–1.615	3.276	0.001	0.283	0.197
	CC vs. GG	Fixed	3.39	0.404	2.025	1.399–2.929	3.744	≤ 0.001	1.000	0.314
	CG vs. GG	Random	59.78	0.008	1.375	1.125–1.681	3.108	0.002	0.210	0.236
	CC + CG vs. GG	Random	62.81	0.004	1.387	1.130–1.703	3.132	0.002	0.152	0.205
	CC vs. CG + GG	Fixed	0.00	0.461	1.767	1.224–2.550	3.042	0.002	0.710	0.445
Asians	C vs. G	Random	61.45	0.008	1.361	1.139–1.627	3.392	0.001	0.348	0.316
	CC vs. GG	Fixed	3.39	0.404	2.025	1.399–2.929	3.744	≤ 0.001	1.000	0.314
	CG vs. GG	Random	61.62	0.008	1.390	1.139–1.697	3.238	0.001	0.348	0.384
	CC + CG vs. GG	Random	64.65	0.004	1.403	1.145–1.719	3.264	0.001	0.251	0.328
	CC vs. CG + GG	Fixed	0.00	0.461	1.767	1.224–2.550	3.042	0.002	0.710	0.445
Chinese	C vs. G	Fixed	64.21	0.025	1.184	0.897–1.563	1.191	0.234	0.806	0.885
	CC vs. GG	Fixed	0.00	0.867	0.972	0.421–2.245	-0.066	0.947	1.000	0.850
	CG vs. GG	Fixed	64.29	0.024	1.224	0.908–1.652	1.326	0.185	0.806	0.882
	CC + CG vs. GG	Fixed	65.10	0.022	1.212	0.900-1.631	1.265	0.206	0.806	0.885
	CC vs. CG + GG	Fixed	0.00	0.872	0.960	0.416–2.215	-0.096	0.924	1.000	0.786
Source of Controls										
PB	C vs. G	Random	61.46	0.075	1.103	0.591–2.060	0.308	0.758	1.000	0.642
	CC vs. GG	Random	60.77	0.110	1.326	0.073–5.977	0.190	0.849	NA	NA
	CG vs. GG	Fixed	34.06	0.219	1.206	0.855–1.701	1.067	0.286	1.000	0.506
	CC + CG vs. GG	Random	51.97	0.125	1.133	0.634–2.024	0.422	0.673	1.000	0.587
	CC vs. CG + GG	Random	58.45	0.121	1.005	0.173–5.829	0.006	0.995	NA	NA
HB	C vs. G	Fixed	45.00	0.162	1.541	1.366–1.739	7.024	≤ 0.001	0.296	0.142
	CC vs. GG	Fixed	39.16	0.193	2.214	1.482–3.308	3.879	≤ 0.001	1.000	0.671
	CG vs. GG	Random	60.38	0.080	1.544	1.218–1.956	3.594	≤ 0.001	0.296	0.421
	CC + CG vs. GG	Random	59.82	0.083	1.578	1.256–1.982	3.913	≤ 0.001	0.296	0.280
	CC vs. CG + GG	Fixed	41.70	0.180	1.888	1.267–2.812	3.126	0.002	1.000	0.759
Genotyping Methods										
TaqMan	C vs. G	Fixed	42.31	0.158	1.493	1.308–1.705	5.928	≤ 0.001	0.734	0.441
	CC vs. GG	Fixed	0.00	0.596	1.751	1.073–2.857	2.243	0.025	0.734	0.897
	CG vs. GG	Random	50.90	0.106	1.500	1.190–1.891	3.435	0.001	0.734	0.295
	CC + CG vs. GG	Random	55.38	0.081	1.505	1.185–1.912	3.354	0.001	0.734	0.357
	CC vs. CG + GG	Fixed	0.00	0.661	1.476	0.908-2.400	1.571	0.116	0.734	0.942
Sequencing	C vs. G	Fixed	40.66	0.185	1.606	1.349–1.911	5.337	≤ 0.001	1.000	0.833
	CC vs. GG	Fixed	0.00	0.704	3.248	1.719–6.136	3.629	≤ 0.001	NA	NA
	CG vs. GG	Random	52.74	0.120	1.641	1.053–2.557	2.189	0.029	1.000	0.308
	CC + CG vs. GG	Fixed	44.85	0.163	1.656	1.357–2.021	4.967	≤ 0.001	1.000	0.807
	CC vs. CG + GG	Fixed	0.00	0.722	2.874	1.528–5.408	3.274	0.001	NA	NA

Abbreviations: I^2^ - Heterogeneity statistic, P_H_ - *P*-value for heterogeneity, OR - Odds Ratio, CI - Confidence Interval, Z_test_ - Z-test statistic, P_OR_ - *P*-value for overall result, PB - Population-Based, HB - Hospital-Based, NA - Not Applicable

#### rs2290692

Table 3 details the association between the ITPKC rs2290692 polymorphism and KD risk. Analysis of pooled data from four studies, involving 1,039 cases and 1,688 controls, shows that this polymorphism is not associated with KD across all five genetic models.

#### rs7251246

The data presented in Table 3 provides a comprehensive analysis of the correlation between the rs7251246 polymorphism and susceptibility to KD. The combined findings from two research studies involving 595 cases and 820 controls do not indicate a clear association between the ITPKC rs7251246 polymorphism and predisposition to KD.

#### rs10420685

The data in Table 3 offers a thorough examination of the relationship between the rs10420685 polymorphism and susceptibility to KD. The collective results from two investigations, encompassing a total of 671 cases and 739 controls, do not suggest a definitive association between the ITPKC rs10420685 polymorphism and the risk of developing KD.

**Table 3:** Summary risk estimates for association of ITPKC rs2290692, rs7251246 and rs10420685 polymorphisms with KD risk

Subgroup	Genetic Model	Type of Model	Heterogeneity		Odds Ratio				Publication Bias	
			**I** ^**2**^ **(%)**	**P**_**H**_	**OR**	**95% CI**	**Z**_**test**_	**P**_**OR**_	**P**_**Beggs**_	**P**_**Eggers**_
rs2290692	C vs. G	Random	75.21	0.007	1.128	0.857–1.483	0.859	0.390	0.734	0.729
	CC vs. GG	Random	76.44	0.005	1.226	699 − 2.150	0.711	0.477	0.308	0.621
	CG vs. GG	Fixed	0.00	0.621	1.254	1.012–1.555	2.069	0.039	1.000	0.494
	CC + CG vs. GG	Fixed	32.39	0.218	1.276	1.043–1.560	2.369	0.009	1.000	0.900
	CC vs. CG + GG	Random	78.01	0.003	1.064	0.653–1.734	0.249	0.804	0.734	0.616
rs7251246	C vs. G	Fixed	0.00	0.833	1.058	0.910–1.229	0.731	0.465	NA	NA
	CC vs. GG	Fixed	0.00	0.837	1.141	0.838–1.555	0.839	0.402	NA	NA
	CG vs. GG	Random	0.00	0.597	1.133	0.871–1.473	0.933	0.351	NA	NA
	CC + CG vs. GG	Random	0.00	0.752	1.132	0.882–1.453	0.976	0.329	NA	NA
	CC vs. CG + GG	Fixed	0.00	0.535	1.029	0.804–1.318	0.228	0.820	NA	NA
rs10420685	C vs. G	Random	81.46	0.020	0.807	0.534–1.221	-1.013	0.311	NA	NA
	CC vs. GG	Fixed	0.00	0.864	0.950	0.605–1.491	-0.224	0.823	NA	NA
	CG vs. GG	Fixed	0.00	0.708	1.053	0.839–1.322	0.446	0.656	NA	NA
	CC + CG vs. GG	Fixed	0.00	0.728	1.036	0.834–1.287	0.318	0.750	NA	NA
	CC vs. CG + GG	Fixed	0.00	0.929	0.925	0.595–1.438	-0.345	0.730	NA	NA

Abbreviations: I^2^ - Heterogeneity statistic, P_H_ - *P*-value for heterogeneity, OR - Odds Ratio, CI - Confidence Interval, Z_test_ - Z-test statistic, P_OR_ - *P*-value for overall result, NA - Not Applicable

### Between-Study heterogeneity

The investigation into between-study heterogeneity regarding the ITPKC rs28493229 polymorphism and KD risk found significant variability across subgroups and genetic models. High heterogeneity was observed, especially in comparisons such as C vs. G (I² = 59.46, PH = 0.008), CG vs. GG (I² = 59.78, PH = 0.008), and CC + CG vs. GG (I² = 62.81, PH = 0.004). In contrast, CC vs. GG showed low heterogeneity (I² = 3.39, PH = 0.404), indicating more uniform effects. In the Asian subgroup, significant heterogeneity persisted for C vs. G (I² = 61.45, PH = 0.008), CG vs. GG (I² = 61.62, PH = 0.008), and CC + CG vs. GG (I² = 64.65, PH = 0.004), while CC vs. GG exhibited low heterogeneity. Among the Chinese population, a fixed effects model indicated notable heterogeneity for C vs. G (I² = 64.21, PH = 0.025) and similar patterns for CG vs. GG (I² = 64.29, PH = 0.024) and CC + CG vs. GG (I² = 65.10, PH = 0.022), with no heterogeneity for CC vs. GG and CC vs. CG + GG. When stratified by control source, population-based studies showed moderate heterogeneity for C vs. G (I² = 61.46, PH = 0.075) and CC vs. GG (I² = 60.77, PH = 0.110), whereas hospital-based studies had lower heterogeneity. The TaqMan genotyping method showed fixed heterogeneity for C vs. G (I² = 42.31, PH = 0.158) and none for CC vs. GG (I² = 0.00), while sequencing consistently displayed lower heterogeneity across models (Table 2). The analysis of other ITPKC polymorphisms (rs2290692, rs7251246, and rs10420685) revealed varying heterogeneity levels. The rs2290692 polymorphism showed substantial heterogeneity in several comparisons (e.g., C vs. G with I² = 75.21%, PH = 0.007), while CG vs. GG showed none (I² = 0.00%, PH = 0.621). The rs7251246 polymorphism presented no heterogeneity across all models, while rs10420685 indicated significant heterogeneity in the C vs. G comparison (I² = 81.46%, PH = 0.020), with other models demonstrating no heterogeneity (Table 3). These findings highlight considerable heterogeneity in associations between specific ITPKC polymorphisms and KD risk, with CC vs. GG genotype comparisons offering more reliable outcomes. Overall, rs2290692 and rs10420685 show notable variability, while rs7251246 provides consistent results without heterogeneity, underscoring the inconsistent impacts of ITPKC variants on KD susceptibility.

### Sensitivity analysis

The sensitivity analysis demonstrated that excluding individual studies did not significantly alter the overall ORs or 95% CIs, confirming the robustness of the meta-analysis results. However, the exclusion of the study by Onouchi et al. (2008) notably affected the variant rs2290692, indicating that this study may disproportionately influence the overall effect estimates. This highlights the necessity for further investigation into the characteristics of the Onouchi study that could explain these discrepancies. While the overall findings are consistent, the sensitivity of rs2290692 to the exclusion of this study underscores the importance of cautious interpretation and the potential impact of individual studies on collective results. Future research should explore factors such as sample size, population characteristics, and methodological differences to enhance the validity of conclusions in the field.

### Publication bias

**Fig. 3:**
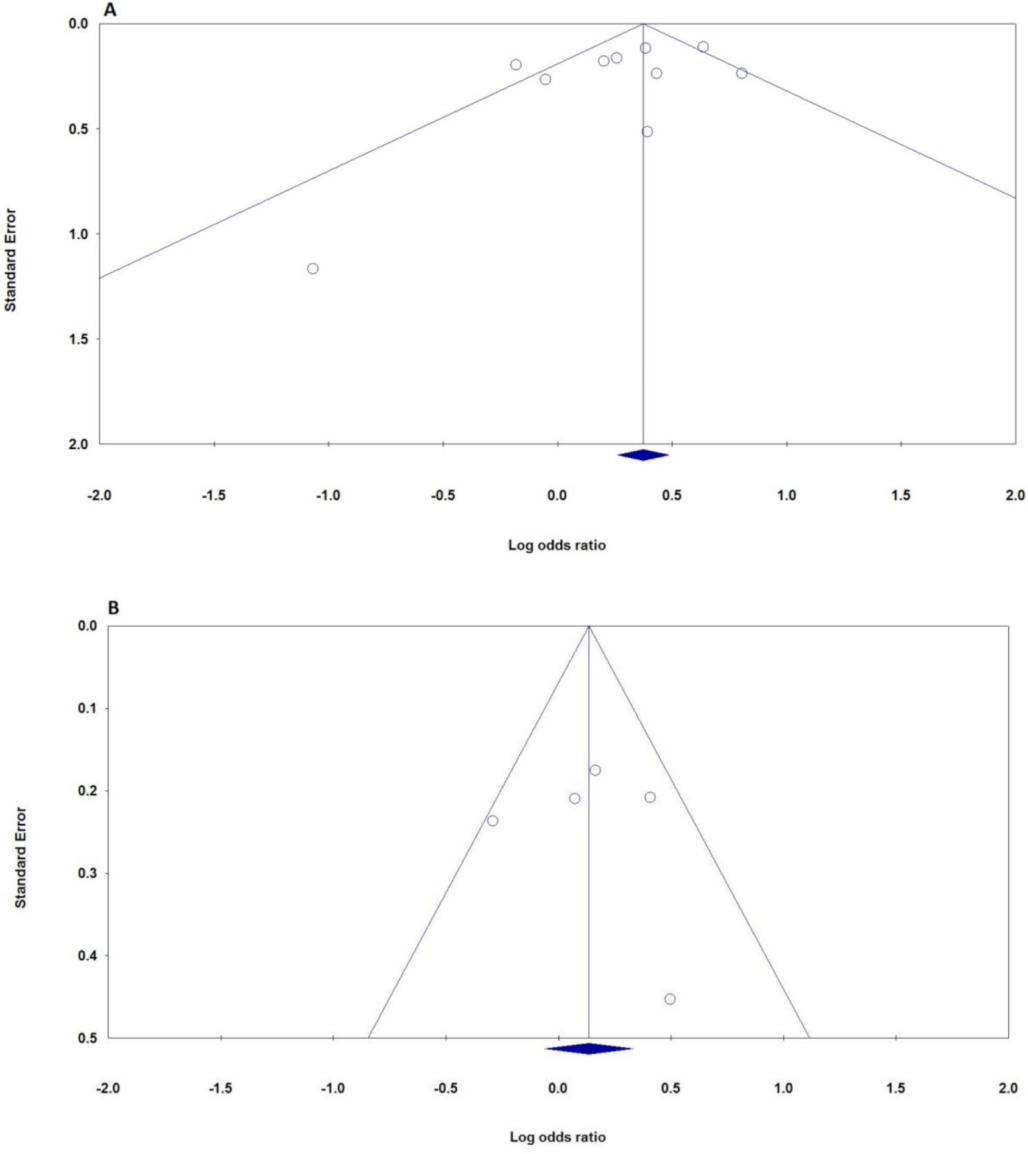
Funnel plots evaluating publication bias for the ITPKC rs28493229 and rs2290692 polymorphisms in relation to KD risk in the overall population. **A**: rs28493229 (allele model: C vs. G); **B**: rs2290692 (heterozygote model: CG vs. GG)

The assessment of publication bias from Tables 2 and 3, particularly the *P*-values from Begg’s test (PBeggs) and Egger’s test (PEggers), provides important insights. For the ITPKC rs28493229 polymorphism’s association with KD, publication bias differed across subgroups and genetic models. The comparison of C and G alleles showed no significant bias, with Begg’s and Egger’s *p*-values of 0.283 and 0.197, respectively. The CC versus GG comparison indicated no bias, with Begg’s *p*-value of 1.000 and Egger’s *p*-value of 0.314. The CG versus GG comparison also showed no bias in a random model, yielding *p*-values of 0.210 and 0.236, respectively. The combined CC + CG versus GG model demonstrated no bias with *p*-values of 0.152 and 0.205; the CC versus CG + GG comparison supported this with *p*-values of 0.710 and 0.445. Ethnic subgroup analyses, particularly within the Asian population, indicated no significant bias, evidenced by Begg’s and Egger’s *p*-values of 0.348 and 0.316, consistent with findings in the Chinese subgroup. In control source stratification, population-based studies showed no bias, while hospital-based studies exhibited variability with *p*-values of 0.296 and 0.142, suggesting moderate bias. Genotyping methods reflected this trend, with TaqMan assays showing a Begg’s *p*-value of 0.734 and sequencing methods yielding a perfect *p*-value of 1.000, indicating no bias. For other ITPKC polymorphisms, such as rs2290692, *p*-values ranged from 0.308 to 0.734, suggesting no significant bias, although rs7251246 and rs10420685 lacked sufficient data for analysis. Figure 3 presents funnel plots for the ITPKC rs28493229 under the allele model (Fig. 3A) and rs2290692 under the heterozygote model (Fig. 3B) related to KD risk in the overall population. Overall, these findings indicate minimal evidence of publication bias in studies of ITPKC polymorphisms and KD risk. While most comparisons across polymorphisms and subgroups showed no significant bias, caution is advised for specific models or stratifications where results varied.

### HWE and MAFs

In evaluating the HWE and MAF among the studies included in the meta-analysis, several key findings emerged. Overall, the MAFs for various SNPs ranged significantly, with rs28493229 (0.040 to 0.155), rs2290692 (0.399 to 0.529), rs7251246 (0.433 to 0.528), and rs10420685 (0.210 to 0.276). The average MAF was approximately 0.317 across all SNPs, indicating a notable diversity in allele frequency. Most studies appeared to comply with HWE, as reflected by the *p*-values ranging from 0.059 to 0.970, although the SNP rs2290692 exhibited exceptions, notably in Bhattarai 2021. Subgroup analyses revealed that Asian studies showed an average MAF of approximately 0.342, with a similar trend in HWE compliance but with specific deviations for rs2290692. In contrast, the Caucasian subgroup was limited to one study from Australia, indicating compliance with HWE but restricting comprehensive evaluation. Overall, while HWE compliance was largely observed, the variability in MAF, particularly in Asian populations, highlights the need for further investigation into genetic associations and clinical significance across diverse ethnic backgrounds.

## Discussion

The ITPKC gene functions as a regulator of calcium channels by phosphorylating IP3, leading to the inhibition of the Ca2+/calcineurin/NFAT signaling pathway, and consequently, a suppressive effect on T cell activation [4]. IP3 acts as a secondary messenger within this pathway, triggering the release of calcium from intracellular stores in the endoplasmic reticulum. This release prompts NFAT nuclear translocation, triggering cytokine release to initiate an immune response and reduce T cell activation, consequently lowering white blood cell IL-2 production [52]. Mutations in the ITPKC gene can disrupt the regulation of T cell activation. Reduced IP3 levels and open calcium channels can cause T cells to become overactivated. NFAT plays a crucial role in the immune system, contributing to blood vessel stabilization, angiogenesis, and endothelial cell inflammation. Research indicates that damage to vascular endothelial cells can elevate NFATc1 levels, along with NFATc3 and inflammatory factors [28]. Moreover, the ITPKC gene can regulate nucleotide-binding oligomerization domain-like receptor protein 3 (NLRP3) inflammation through Ca2 + mobilization. Activation of the NLRP3 inflammasome is the innate immune response to various stimuli. Factors triggering NLRP3 inflammasome activation include high mobility group protein B1/glycation end product receptor/Cathepsin B signal, K + efflux, and Ca2 + signal. Studies suggest that NLRP3 inflammasome activation correlates with IL-1β and IL-18 circulation in children with KD, associated with increased ring protein levels. Multiple studies have linked the rs28493229 variant of the ITPKC gene to an increased susceptibility to KD and the development of coronary artery lesions (CAL) in populations from China, Japan, and India. Its influence on KD risk differs by ethnicity; it elevates risk in Japanese and Taiwanese populations, but consistent associations are absent in North Indian groups [53, 54]. This polymorphism primarily affects ITPKC function by altering the splicing efficiency of intron 1, resulting in reduced levels of mature ITPKC mRNA. This decrease impairs the normal regulation of calcium signaling pathways, particularly the calcineurin/NFAT pathway, where ITPKC acts as a negative regulator by phosphorylating IP3 to limit T cell activation and cytokine production [4]. Diminished ITPKC expression due to the rs28493229 polymorphism can lead to increased signaling through these pathways, resulting in heightened immune activity and elevated cytokine release [34]. The C allele associated with this polymorphism is linked to lower gene expression and potentially higher intracellular calcium concentrations via IP3-mediated pathways, which may exacerbate inflammatory responses and contribute to conditions like KD. Notably, this variant has been found to be expressed in IVIG-unresponsive KD patients in Japan, correlating with significantly higher IVIG responses. The rs28493229 polymorphism has been shown to inhibit KD symptoms and is associated with acute phase KD with Bacille Calmette-Guérin (BCG) scar reactivation [55]. Furthermore, the C allele of rs28493229 is associated with a higher risk of developing CALs, underscoring the role of ITPKC variants in KD-related vascular complications [34]. While the link between rs28493229 and KD susceptibility highlights the importance of immune regulation and calcium signaling, existing research has limitations in data reliability and study quantity, necessitating further investigation, especially in subgroup analyses [34, 45]. However, the aggregated data from these studies remain inconclusive due to these limitations, and the association has only been evaluated under the allele genetic model without conducting subgroup analyses.

Studies indicate a strong association between ITPKC rs28493229 polymorphism and an increased risk of KD, especially in the general population and specific ethnic groups. A meta-analysis on Indian children also confirms this link, emphasizing the importance of the CG + GG genotype of SNP rs2290692 in the ITPKC gene [51]. Moreover, research on Koreans shows that SNP rs28493229 in ITPKC is connected to KD and coronary artery complications, highlighting the influence of genetic variations on disease development and outcomes. In our meta-analysis of ten studies involving 2771 cases and 5357 controls, we found a significant correlation between the rs28493229 genetic variation and heightened KD susceptibility across various genetic models. Similarly, a study by Lou et al. (2012) analyzing seven case-control studies with 3,821 cases and 12,802 controls, revealed a significant link between the C allele of the rs28493229 polymorphism and an increased KD risk [37]. Furthermore, a meta-analysis by Bhattarai et al. (2021) on the CC genotype of rs28493229 suggested a 1.5-fold higher susceptibility for individuals with this genotype to develop KD [51]. Moreover., Khor et al. (2011) conducted a GWAS that revealed a significant association between the rs28493229 locus and KD risk, with a *p*-value of 1.68 × 10^(-12) and an OR of 1.52. This finding indicates that individuals carrying the risk allele for rs28493229 have a 52% increased likelihood of developing KD compared to those without the allele. The strong association points to a potential role of ITPKC in the pathophysiology of KD, likely linked to its involvement in immune regulation and signaling pathways. These results align with previous research identifying genetic factors influencing the disease, underscoring the importance of genetic predisposition in KD susceptibility [19]. Furthermore, understanding how rs28493229 interacts with the immune response could shed light on the mechanisms underlying the disease’s clinical manifestations and its treatment, particularly regarding the effectiveness of intravenous immunoglobulin therapy.

Our analysis of four studies, totaling 1,039 cases and 1,688 controls, found no link between rs2290692 polymorphism and KD risk. Peng et al. (2012) studied five ITPKC variations, including rs28493229, in 223 Han Chinese KD patients and 318 controls, revealing no significant differences in the C allele, CC genotype, or C carriers of rs28493229. However, patients exhibited higher frequencies of the C allele, CC genotype, and C carriers for rs2290692 compared to controls, a pattern also observed in those with and without CALs. Additionally, the GC haplotype of rs28493229 and rs2290692 was more prevalent in patients [55]. Similarly, Kuo et al. (2014) confirmed a link between rs2290692 and CAL formation in Taiwanese KD patients [56]. Bhattarai et al. (2021) reported no significant association of ITPKC rs28493229 and rs2290692 polymorphisms with KD susceptibility among North Indian children. Their findings indicated that while rs28493229 does not correlate with KD susceptibility, the combined genotype of rs2290692 does [51]. The impact of ITPKC polymorphisms on KD varies among racial and ethnic groups, highlighting that the relationship between phenotype and genotype in KD may differ across ethnicities.

Studies have shown that the rs2290692 and rs28493229 loci are in complete LD (D’=1.0) across various ethnic groups, indicating that the association of one polymorphism could account for the presence of the other [48, 57]. This genetic linkage suggests that these variants may not independently influence KD susceptibility but instead represent a shared genetic framework affecting the disease’s pathogenesis. Research has established a connection between the ITPKC gene and KD susceptibility, especially in Indian [51], Japanese [46], and Han Chinese [55] children. Recent investigations into the specific SNPs rs284903229 and rs2290692 within the ITPKC gene revealed that in a North Indian cohort [51], the combined genotype of rs2290692 was significantly associated with KD susceptibility (*p* = 0.015, ORs = 4.1), while no significant association was found for rs284903229 alone or its single alleles. This suggests differing individual contributions to KD risk, despite the complete LD. The rs2290692 polymorphism has been validated in genome-wide studies [58] and meta-analyses [51], highlighting a strong genetic predisposition to KD, particularly within Han Chinese populations. The functional implications of these polymorphisms extend beyond association, as they may influence immune responses in KD, with genetic factors in the ITPKC pathway potentially modulating T-cell activation and cytokine production, which are crucial in the inflammatory process of KD [59]. Consequently, the presence of these polymorphisms may alter immune responses, increasing susceptibility to KD and its complications, such as coronary artery lesions. While both SNPs are inherited together more often than expected, this does not imply equal contributions to KD susceptibility; instead, the association of rs2290692 suggests it may be the primary genetic risk factor in certain populations, while rs284903229 does not exhibit the same effect.

Our analysis of data from two studies with a total of 595 cases and 820 controls indicates that the ITPKC rs7251246 polymorphism is not associated with susceptibility to KD. Kuo et al. (2014) conducted a thorough investigation on the correlation between seven tagging SNPs of ITPKC and KD risk in a Taiwanese population consisting of 381 KD patients and 569 controls. Their findings revealed a significant connection between rs7251246 in ITPKC and the development of CAL. Haplotype analysis further supported this link in patients with CAL and aneurysm formation. This research was the first to establish that the SNP rs7251246 in ITPKC is linked to the severity of KD [56]. Subsequently, Liu et al. (2023) studied 262 children as controls and 221 children with KD to delve into the relationship between the ITPKC rs7251246 polymorphism, KD susceptibility, and CAAs formation. They discovered that although the ITPKC rs7251246 T > C polymorphism was not significantly linked to KD susceptibility, it was notably associated with CAA risk in children with KD. Male children with the rs7251246 CT/TT genotype had a significantly lower thrombosis risk. Furthermore, children with KD, especially those with CAA, showed notably lower levels of ITPKC mRNA compared to healthy children. In children with CAA who experienced thrombosis, ITPKC mRNA levels were decreased. Children with KD and the CC genotype exhibited reduced mRNA levels of ITPKC [44]. Moreover, research has revealed that the rs10420685 polymorphism is linked to KD susceptibility and severity, particularly in relation to CAL and aneurysm formation [2]. Furthermore, interactions between ITPKC polymorphisms, including rs10420685, and other genes like solute carrier 11a1 (SLC11A1) have been found to influence the risk of KD and the erythema of the BCG injection site [1]. However, the collective results from two investigations encompassing a total of 671 cases and 739 controls do not appear to suggest a definitive association between the ITPKC rs10420685 polymorphism and the risk of developing KD.

### Limitations

To the best of our knowledge, this is the first meta-analytic investigation into the association between the rs2290692, rs7251246, and rs10420685 polymorphisms and the development of KD. It is also the most comprehensive meta-analysis for rs28493229, based on an extensive database search. However, several limitations should be considered. Firstly, some studies had small sample sizes, which could affect the generalizability of the results. Future research with larger and more diverse samples would strengthen the evidence base and lead to more robust conclusions. Additionally, the methodologies employed across the studies varied significantly, making it challenging to compare results directly. Standardizing research methods and protocols is essential to enhance the consistency and reliability of future meta-analyses in this area. Secondly, the literature search was restricted to English and Chinese sources, potentially overlooking relevant studies published in other languages and introducing a selection bias. Future research should aim to broaden language coverage to ensure a more thorough review of existing literature. Thirdly, most studies focused primarily on Asian populations, which may introduce ethnicity bias. It’s crucial for future studies to include participants from Caucasian and African backgrounds to address this issue effectively. By expanding the scope of investigations and incorporating data from diverse populations, researchers can gain a more comprehensive understanding of the genetic factors influencing susceptibility to KD. Moreover, the limited number of studies available for subgroup analyses may have reduced the statistical power to detect a link between the ITPKC rs28493229 polymorphism and KD risk. Subgroup analyses for rs2290692 and rs7251246 polymorphisms were not possible due to the scarcity of studies. Future research should prioritize increasing the number of studies in these areas to enhance statistical power and provide stronger conclusions regarding the relationship between ITPKC polymorphisms and KD risk. Finally, factors such as age, gender, family history, and ethnicity may have influenced the results. Exploring the combined interactions of relevant genes is crucial for a comprehensive understanding of the etiology of KD. Additionally, future research directions could significantly benefit from incorporating functional studies aimed at confirming the mechanistic role of ITPKC variants in KD pathogenesis. With these minor corrections and clarifications on limitations, the paper can contribute meaningfully to the understanding of genetic susceptibility in KD.

### Future directions

Future research on KD should prioritize functional studies to clarify the mechanistic role of ITPKC variants, particularly polymorphisms like rs28493229 and rs2290692, in the disease’s pathogenesis. These studies should investigate how these genetic changes impact calcium signaling pathways, T cell activation, and cytokine production. Additionally, researchers should examine the influence of environmental factors and their interactions with genetic predispositions across different ethnic groups, conducting subgroup analyses to identify population-specific risk factors and the varying effects of these variants on KD susceptibility and severity. Furthermore, exploring the relationship between ITPKC SNPs and other immune regulatory pathways, especially regarding the development of coronary artery lesions in KD patients, could lead to targeted therapies to reduce severe complications. Large-scale genetic studies, including genome-wide associations focusing on ITPKC’s influence on KD, are also necessary to bolster the evidence linking genetic variants to KD susceptibility in diverse populations. Longitudinal studies could provide insights into how these genetic factors affect immune responses and treatment outcomes over time, particularly in relation to the responsiveness to intravenous immunoglobulin therapy. Overall, a deeper understanding of the biological processes involved is essential to substantiate the connection between ITPKC variants and Kawasaki Disease with experimental evidence.

## Conclusions

Quantitative data synthesis indicates a significant association between the rs28493229 polymorphism in the ITPKC gene and increased risk of KD, particularly among Asians, though the Chinese subgroup did not show a notable risk. In contrast, the rs2290692, rs7251246, and rs10420685 polymorphisms did not demonstrate clear associations with KD risk in various studies and models. These findings underscore the importance of specific genetic variants in KD susceptibility, as well as the impact of demographic and methodological factors on observed associations. Further research is necessary to elucidate the mechanisms involved and evaluate the effect of genetic variability on KD risk across different populations. Future studies should also prioritize functional analyses of ITPKC variants and explore interactions among related genes to better understand the origins of KD. Enhanced research methodologies will improve these findings and provide deeper insights into the genetic factors influencing KD susceptibility.

## Electronic supplementary material

Below is the link to the electronic supplementary material.

Supplementary Material 1

## Data Availability

The datasets generated during and/or analyzed during this study are the corresponding author on reasonable request.

## Abbreviations

- KD: Kawasaki Disease
- CAL: Coronary Artery Lesions
- IVIG: Intravenous Immunoglobulin
- ASA: Acetylsalicylic Acid (Aspirin)
- IKD: Incomplete Kawasaki Disease
- GWAS: Genome-Wide Association Studies
- SNP: Single Nucleotide Polymorphism
- WGS: Whole Genome Sequencing
- IP3: Inositol 1,4,5-trisphosphate
- IP4: Inositol 1,3,4,5-tetrakisphosphate
- NFAT: Nuclear Factor of Activated T-cells
- MeSH: Medical Subject Headings
- PMC: PubMed Central
- CINAHL: Cumulative Index to Nursing and Allied Health Literature
- EKB: Egyptian Knowledge Bank
- CNKI: Chinese National Knowledge Infrastructure
- CBD: Chinese Biomedical Database
- CHBD: Circumpolar Health Bibliographic Database
- CAOD: China/Asia On Demand
- ICI: Indian Citation Index
- CMCI: Chinese Medical Citation Index
- CMCC: Chinese Medical Current Contents
- VIP: VIP Information Consultancy Company
- CI: Confidence Interval
- CMA: Comprehensive Meta-Analysis
- HWE: Hardy-Weinberg Equilibrium
- ITPKC: Inositol Trisphosphate 3-kinase C
- MAF: Minor Allele Frequency
- NOS: Newcastle-Ottawa Score
- OR: Odds Ratio
- p: *p*-value
- RFLP-PCR: Restriction Fragment Length Polymorphism-Polymerase Chain Reaction
- SNaPshot: Single Nucleotide Polymorphism analysis
- W: Wild type allele
- M: Mutant allele
- I^2^: I-squared statistic
